# The Nation's Fund for Nurses

**Published:** 1921-03-26

**Authors:** 


### The Nation's Fund for Nurses.

### THE PROGRESS OF ITS TWO OBJECTS.

The Hon. Sir Arthur Stanley was in the chair at a meeting of the Nation's Fund for Nurses held at the Royal Automobile Club on March 18. The meeting, which was attended by members of the Council of the College of Nursing, the British Women's Hospital Committee, and the Council and Executive and Tribute Committees of the Nation's Fund, was convened in order that any difficulties which had presented themselves with regard to the report of the British Women's Hospital Committee might be brought forward and dealt with.

Sir Arthur gave an instructive sketch of the origin of the Nation's Fund for Nurses, which was inaugurated by the British Women's Hospital Committee in 1917 with two definite and distinct objects—(1) the endowment of the College of Nursing, and (2) the creation of a Benevolent or Tribute Fund for the relief of individual nurses during sickness and disablement. The British Women's Hospital Committee conducted the affairs of both funds with marked success and ability from that date till the end of 1919, when they were taken over by a Council composed of ladies who have shown special interest in the welfare of nurses throughout the country, including the whole of the Council of the College of Nursing. Up to December 31, 1919, a total of £148,915 had been raised for the Nation's Fund for Nurses, of which £78,401 was earmarked for the Tribute Fund and £18,636 for the College of Nursing Endowment and Scholarship Fund. The cost of raising this large sum was under 5 per cent. The balance sheet is published in our issue of February 26, 1921, and on pp. 479-80 of our issue of February 19 we give details of the figures; we do not therefore recapitulate what Sir Arthur Stanley said under this head at the meeting on the 18th inst. It is, however, desirable to emphasise the fact that the £50,000 contributed to the Tribute Fund by the British Red Cross Society was given with the express stipulation that the money should be vested in trustees, and only the income derived therefrom should be distributed for the relief of nurses; 163 individuals have been so assisted.

The total aimed at by the inaugurators of the Nation's Fund was £100,000 for each of its two objects. It is understood that the report for 1920, shortly to be issued, will show that the Tribute Fund has attained this amount. The College Endowment Fund falls considerably short of it, but Lord and Lady Cowdray's munificent gift of a College building has greatly reduced the charge on capital which the Endowment Fund would have had to face, whilst it has provided College members with a far more worthy building than would otherwise have been possible.

Dame Sidney Browne proposed, and Lady Sheffield seconded, a vote of thanks to the British Women's Hospital Committee for all they had achieved for nurses in spite of many difficulties and considerable unpleasantness.

